# The role of fecal microbiota transplantation in type 2 diabetes mellitus treatment

**DOI:** 10.3389/fendo.2024.1469165

**Published:** 2024-12-13

**Authors:** Huimei Wang, Shuo Li, Luping Zhang, Nan Zhang

**Affiliations:** Department of Gastroenterology, The First Hospital of Jilin University, Changchun, China

**Keywords:** type 2 diabetes mellitus, fecal microbiota transplantation, gut microbiota, dysbiosis, metabolites

## Abstract

In contemporary microbial research, the exploration of interactions between microorganisms and multicellular hosts constitutes a burgeoning field. The gut microbiota is increasingly acknowledged as a pivotal contributor to various disorders within the endocrine system, encompassing conditions such as diabetes and thyroid diseases. A surge in research activities has been witnessed in recent years, elucidating the intricate interplay between the gut microbiota and disorders of the endocrine system. Simultaneously, fecal microbiota transplantation (FMT) has emerged as a focal point, garnering substantial attention in both biomedical and clinical spheres. Research endeavors have uncovered the remarkable therapeutic efficacy of FMT across diverse diseases, with particular emphasis on its application in addressing type 2 diabetes mellitus (T2DM) and associated com-plications. Consequently, this manuscript accentuates the intimate connection between the gut microbiota and disorders within the endocrine system, with a specific focus on exploring the potential of FMT as an intervention in the therapeutic landscape of T2DM and its complications. Furthermore, the article scrutinizes concerns inherent in treatment modalities centered around the gut microbiota, proposing viable solutions to address these issues.

## 1 Introduction

Type 2 diabetes mellitus (T2DM) is a prevalent metabolic chronic disease characterized by insulin resistance and abnormally elevated blood glucose levels (1). Currently, the global number of adults with T2DM exceeds 400 million, and this figure continues to rise (2). This has led to a substantial burden of mortality and disability worldwide, contributing to a sustained increase in healthcare expenditures across nations (3). Notably, diabetic patients with complications incur significantly higher healthcare costs and rates of mortality and disability compared to those without complications (4). Diabetes has numerous associated complications, and all current medications targeting these complications come with certain side effects. The introduction of new treatment strategies is highly necessary and imminent.

Scientific investigations have revealed that the structural resemblance between human cells and bacteria is noteworthy (5). Specifically, the gut microbiota, constituting the most intricate and diverse microbial community within the human body, is commonly denoted as the “second genome” of humans within the bacterial system. Despite undergoing alterations in composition and structure over time, the human gut microbiota maintains a certain level of dynamic stability (5, 6). Mounting evidence underscores the pivotal role of the gut microbiota in host metabolism, immunity, and even behavior, achieved through the breakdown of food or host components and the subsequent generation of novel compounds or metabolites (7, 8). This reciprocal influence between the host and the microbial community establishes a bidirectional interplay characterized by mutual interactions between bacteria and the host, collectively shaping host performance (9). Fecal microbiota transplantation (FMT), a recent focal point of research, has exhibited therapeutic promise in addressing a spectrum of conditions, including metabolic syndrome, autoimmune disorders, and neurologic diseases (10, 11).

## 2 Association between gut microbiota and T2DM

Type 2 diabetes mellitus (T2DM) is characterized by both tissue-specific insulin resistance and dysfunction of pancreatic β-cells. The intricate local mechanisms contributing to T2DM involve metabolic signaling, alterations in mitochondrial metabolism, oxidative stress, endoplasmic reticulum stress, and localized inflammation (12, 13). Furthermore, the gut microbiota plays a pivotal role in the manifestation of T2DM.

### 2.1 Mechanisms of altered gut microbiota in T2DM

Microbial communities constitute the largest “ecosystem” within the human body, evolving concomitantly with human development (14). In healthy adults, the gut microbiota comprises approximately ten times more microbial cells than human cells, with genomes that are a hundred times more abundant (15). The microbiota is fundamentally involved in the development of the immune system, defense against pathogenic microorganisms, digestion of exogenous substances, and the regulation of metabolism (16).

From the esophagus to the rectum, variations in bacterial diversity, abundance, and quantity occur due to genetic predisposition, dietary factors, antibiotic use, and challenging Clostridioides difficile infections, resulting in alterations to the microbial community and dysbiosis (17, 18). Changes in the gut microbiota are associated with host oxidative stress and inflammatory responses. Elevated blood glucose levels can lead to intestinal damage characterized by tight junction protein degradation, compromising barrier integrity (19). Hyperglycemia damages tight and adherens junctions, leading to barrier disruption. This disruption contributes to dysregulation of the gut microbiota, weakening the intestinal clearance of toxic metabolites and increasing the abundance of pathogenic bacteria (20). Intestinal bacteria, entering the bloodstream through the compromised intestinal barrier, stimulate the immune system (21).

**Figure 1:**
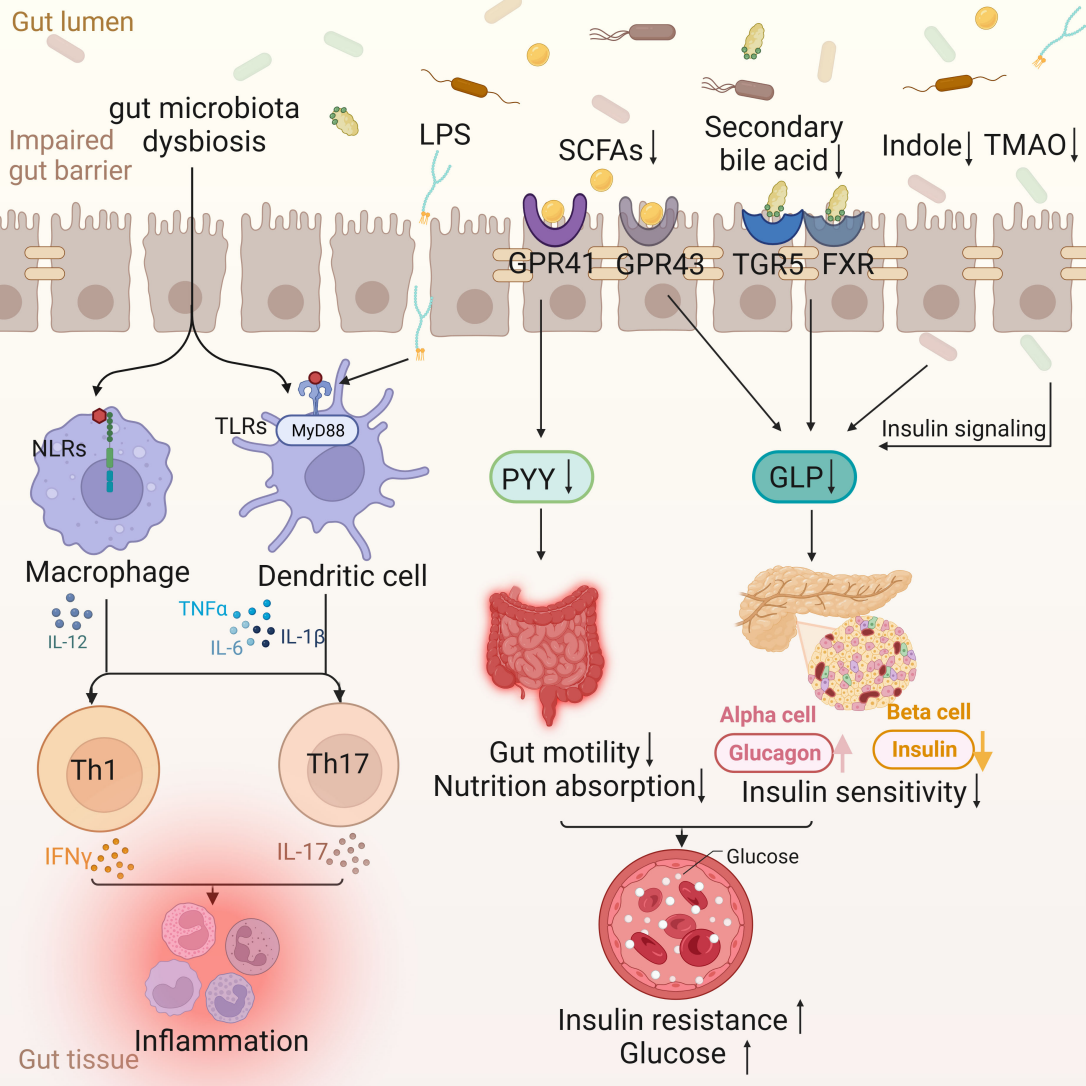
Intestinal changes in T2DM. The disordered gut microflora enters the gut tissue through the impaired gut barrier, activating the nucleotide oligomerization domain (NOD)-like receptors (NLRs) signaling pathway of macrophages and the MyD88-dependent TLRs signaling pathway of dendritic cells, and jointly activating Th 1 and Th 17, leading to the occurrence of inflammation. Lipopolysaccharides (LPS) is also involved in inflammation through Toll-like receptors (TLRs). The decrease of Short-chain fatty acids (SCFAs) leads to the decrease of peptide YY (PYY) through G protein-coupled receptor (GPCR) 41, which in turn leads to the decrease of gut motility and nutrient absorption function. At the same time, the decrease of SCFAs, secondary bile acids, indole and Trimethylamine N-Oxide (TMAO) can lead to the decrease of glucagon-like peptide (GLP) and insulin sensitivity through different ways, and then lead to insulin resistance and blood sugar increase.

Intestinal barrier, comprising immune, biological, mechanical, and mucous barriers, segregates the host from microorganisms in the intestinal lumen, limiting their movement (22). An intact mucosal barrier prevents the migration of microorganisms and their products into the bloodstream (23). Compromised barriers result in heightened intestinal permeability, facilitating the entry of bacteria and their byproducts into the bloodstream. This process activates the mononuclear phagocyte system, triggering the release of inflammatory factors like IL-6 and TNF-α, culminating in chronic low-grade inflammation (24). Furthermore, lipopolysaccharide (LPS) can traverse the intestinal epithelium through compromised tight junctions or chylomicrons (25). In diabetic patients, increased intestinal permeability may be associated with inflammatory responses, where LPS, an influential endotoxin from Gram-negative bacterial cell walls, initiates inflammatory mechanisms by binding to CD14 and Toll-like receptor 4 (TLR4) on macrophage surfaces (26). Molecular proteins such as c-Jun N-terminal kinase (JNK) and p38 mitogen-activated protein kinase (MAPK) regulate the impact of inflammation on insulin signaling (27). Consequently, compromised intestinal barrier integrity and immune imbalance contribute to the development of T2DM (**Figure 1**).

### 2.2 Gut microbiota inT2DM

#### 2.2.1 Alterations and effects of gut microbiota in T2DM

The healthy gut microbiota predominantly consists of anaerobic bacteria, categorized into six phyla: Firmicutes, Bacteroidetes, Proteobacteria, Actinobacteria, Fusobacteria, and Verrucomicrobia (28).

Modifications in the gut microbiota influence the metabolism of the primary intracellular antioxidant, glutathione (GSH), within the host organism (29). Interactions between intestinal epithelial cells and specific symbiotic bacteria lead to the rapid generation of reactive oxygen species (ROS) in host cells, impacting the activation of oxidative stress markers like Nox2 and redox signaling transduction (30). Intestinal inflammation elevates gut permeability, setting off a cascade of events, including infections and systemic inflammation (31, 32).

**Figure 2:**
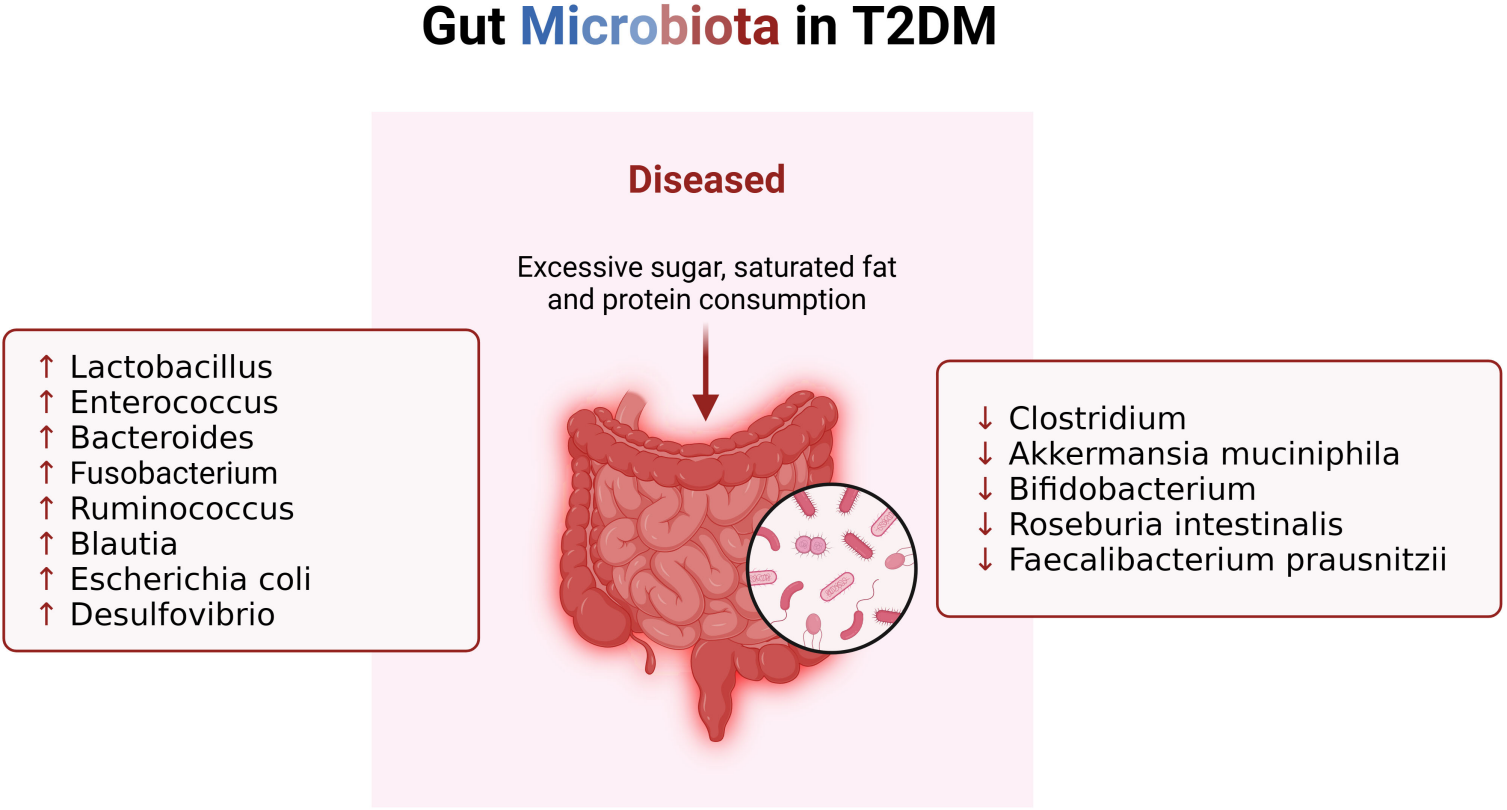
Changes of gut microbiota in patients with T2DM. When T2DM patients consume excessive sugar, saturated fat and protein, their gut microbiota changes, resulting in imbalance of immune system.

A distinctive feature of gut microbiota imbalance in T2DM is the disproportionate ratio between Bacteroidetes and Firmicutes, coupled with a significant reduction in carbohydrate membrane transport and butyric acid biosynthetic function (33, 34) (**Figure 2**). In comparison to their healthy counterparts, T2DM patients exhibit an increase in the abundance of Lactobacillus species and a decrease in Clostridium species in the intestinal tract (28). These bacterial shifts play a role in glucose homeostasis by outcompeting pathogens and reinstating intestinal equilibrium (35). Lactobacillus has been shown to lower blood glucose and body weight while mitigating insulin resistance in high-fat diet-induced T2DM mice (36). Lactobacillus species, particularly Lactobacillus casei, Lactobacillus reuteri, Bifidobacterium bifidum, and Streptococcus thermophiles, can enhance host gut microbial structure and reduce intestinal permeability when consumed in sufficient quantities (37). Another group of bacteria potentially sensitive to insulin is Akkermansia muciniphila, which, when safely ingested, can improve metabolic abnormalities in obese individuals (38). Research indicates that individuals with type 2 diabetes mellitus (T2DM) who are prescribed metformin demonstrate a heightened relative abundance of Akkermansia muciniphila and other microbial groups that produce short-chain fatty acids (SCFAs), in contrast to non-diabetic counterparts (39). This observed phenomenon aligns with findings in human studies, where the increased presence of Akkermansia muciniphila has been associated with the enhanced anti-diabetic effects of metformin in diet-induced obese mice (40). Conversely, T2DM patients exhibit significantly reduced levels of Roseburia intestinalis and Faecalibacterium prausnitzii, both recognized producers of butyrate (41). A randomized double-blind clinical trial has indicated that the incorporation of a freshwater fish-based diet can ameliorate liver fat deposition and other metabolic phenotypes, potentially through the augmentation of fecal Roseburia intestinalis (42). Additionally, a negative correlation has been identified between Bacteroides and T2DM, with diminished levels of specific strains such as Bacteroides 20-3, Bacteroides vulgatus, and Bacteroides intestinalis in individuals with T2D (43). During hyperglycemia, Enterococci increase, while sulfur-producing bacteria such as Bifidobacterium vulgatus decrease, exacerbating diabetes (44). Reduced Bifidobacterium levels lead to elevated plasma endotoxin levels and increased pro-inflammatory cytokines (45). In a study involving 36 adult subjects, healthy individuals had significantly higher levels of Bifidobacterium, whereas T2DM patients had higher levels of Lactobacillus, supporting this conclusion (46). Observational data reveals an elevation in the levels of potentially pathogenic bacteria, notably Escherichia coli and Desulfovibrio, in individuals diagnosed with diabetes (47). Fusobacterium, Ruminococcus, and Blautia consistently emerge as bacterial taxa exhibiting a positive correlation with type 2 diabetes mellitus (T2DM) (48). Furthermore, an augmented abundance of Rikenellaceae has been linked to heightened stress levels and inflammatory responses (49). Muribaculaceae, identified as a beneficial bacterium, plays a pivotal role in positively influencing intestinal energy metabolism while concurrently regulating host blood glucose and lipid levels (50). Clinical studies further reveal that increased levels of Phascolarctobacterium and Bacteroides stercoris are associated with improved insulin sensitivity, linking this metabolic benefit to the success of intestinal endocrine function, gut microbiota, and donor microbial engraftment (51). Moreover, Bacteroides fragilis, a fragile mimic bacterium, is known to generate polysaccharide A, which actively promotes the synthesis of pro-inflammatory cytokines, such as IL-12, thereby fostering Th1 activation (52). The genetic information inherent in these microorganisms correlates with oxidative stress, establishing a direct linkage between alterations in the intestinal microbiota composition and the inflammatory status observed in individuals with T2DM (53).

Additionally, the use of antibiotics significantly alters the gut microbiota, which may affect diabetes control. Hodoşan et al. (54) reported high rates of antibiotic prescriptions, raising concerns about antibiotic resistance and metabolic effects. Păduraru et al. (55)emphasized the clinical consequences of antibiotic use. It is also essential to explore how these factors influence fecal microbiota transplantation and diabetes management.

#### 2.2.2 Gut microbiota influences immunity in T2DM patients

Dysbiosis has been observed to precipitate an immune system imbalance. The gut microbiota and their metabolic byproducts play a pivotal role in sustaining equilibrium within the T-helper 17/regulatory T cells (Th17/Treg) and gut-associated lymphoid tissue (GALT) systems (56). This intricate regulation extends to the promotion of innate lymphoid cells, natural killer cells, cytotoxic and non-cytotoxic cells, as well as helper lymphocytes, with metabolites like tryptophan being involved in this process (57). Innate lymphoid cells (ILCs) represent crucial components of innate immunity, contributing by producing both regulatory and pro-inflammatory cytokines to support tissue repair, immunity, and inflammation. Notably, research has uncovered elevated levels of ILCs1 in individuals diagnosed with T2DM, which correlates with an increased susceptibility to diabetes. Conversely, an increase in ILC2s contributes to improving glucose homeostasis and preventing insulin resistance (58). Additionally, IL-12, as an immune regulatory factor, plays a crucial role in T2DM and its complications. Its signaling, coupled with receptor binding on pancreatic beta cells, triggers pro-inflammatory cytokines and induces cell apoptosis (59). TLR4 activation inhibits insulin function by triggering signal cascades and transcription factors (such as MyD88, TIRAP, TRIF, IKK, and JNK) involved in innate immune responses. TLR4 is widely expressed in cells and promotes inflammation by recognizing damage-associated molecular pattern proteins (DAMPs) (60, 61), ultimately leading to the development of insulin resistance. Another investigation reported that MyD88, a Toll-like receptor (TLR) adapter, possesses the capacity to modulate glucose and lipid metabolism by inducing alterations in the gut microbiota composition (62).

#### 2.2.3 Impact of gut microbiota metabolites

Microbial metabolites, including short-chain fatty acids (SCFAs), secondary bile acids, trimethylamine N-oxide (TMAO), and indole-3-propionic acid (IPA), play a crucial role in either ameliorating or exacerbating T2DM through signaling mediated by their respective receptors (63).

In the human intestinal tract, SCFAs, produced through carbohydrate fermentation, act as substrates for lipid and glucose production (63). Common SCFA-producing bacteria, such as Ruminococcaceae (cluster IV) and Eubacterium (Cluster XIVa) within the Clostridia order, play a pivotal role in this process (64). SCFA receptors, members of the G-protein coupled receptor (GPCR) family, like GPR41 and GPR43, execute crucial physiological functions upon activation by SCFAs, including regulation of fat and glucose metabolism and the production of peptide YY (PYY) (65). The latter, in turn, regulates intestinal motility and nutrient absorption, modulating adipose-insulin signal transduction, leading to reduced energy expenditure and insulin secretion (66, 67). Intestinal SCFAs contribute to reducing inflammation in adipocytes by stimulating the production of glucagon-like peptide 1 (GLP-1), thereby suppressing nuclear transcription factor NF-κB activity. A decrease in GLP-1 levels results in an increase in the concentration of inflammatory factors (such as IL-8 and TNF-α) released by neutrophils and macrophages (64, 68).

The gut microbiota metabolizes primary bile acids into secondary bile acids through bile acid brine hydrolysis and 7α-dehydrogenase (69). Activation of bile acid receptors, including G protein-coupled bile acid receptor 5 (TGR5) and farnesol X receptor (FXR), regulates the secretion of incretin hormone GLP-1 (70, 71). Some secondary bile acids also maintain intestinal barrier function, preventing excessive growth of intestinal bacteria and migration into host cells (72).

Gut bacteria contribute to the formation of trimethylamine (TMA) from choline, transported to the liver, where it is converted into trimethylamine N-oxide (TMAO), exhibiting atherogenic properties (56). Choline, a crucial nutrient for lipid metabolism and the production of very low-density lipoproteins (VLDLs) in the liver, plays a significant role in the occurrence and maintenance of T2DM, correlating with substantial risks for other metabolic syndromes (73).

Indole-3-propionic acid (IPA), endogenously derived from tryptophan by the gut microbiota, is absorbed by intestinal epithelial cells and enters the bloodstream (74). IPA improves glucose metabolism and possesses antioxidant and anti-inflammatory effects. Plasma IPA may serve as a potential biomarker for diabetes and play a protective role by maintaining β-cell function (75).

## 3 FMT and T2DM

### 3.1 Clinical management of T2DM

Current approaches to treating T2DM primarily involve lifestyle modifications, oral medications, injectable drugs, and insulin therapy (76). Diverse diets, such as the Mediterranean diet, low-carbohydrate/high-protein diet, vegetarianism, and veganism, have proven effective in improving metabolic conditions and managing T2DM. Adopting a healthy lifestyle with a balanced, antioxidant-rich diet and regular exercise can significantly delay and/or prevent disease progression (77). The pharmacological arsenal for T2DM includes metformin, sulfonylureas, DPP-4 inhibitors, GLP-1 receptor agonists, and SGLT-2 inhibitors. Beyond targeted medications like GLP-1 agonists and SGLT-2 inhibitors, clinical attention is focused on substantial blood sugar and weight control. Ongoing efforts involve the development of personalized and combination therapies tailored to different genders, ages, and body types (78).

### 3.2 FMT for T2DM treatment

The effectiveness of fecal microbiota transplantation (FMT) in treating Clostridioides difficile infection (CDI) has been substantiated, addressing 80-90% of recurrent CDI cases unresponsive to antibiotics. Currently, FMT is considered the standard treatment in guidelines for managing recurrent CDI (79). FMT proves to be a safe therapeutic approach, with mild clinical symptoms being the most common adverse reactions observed in all existing clinical cases, including diarrhea, gastrointestinal spasms, nausea, abdominal distension, flatulence, constipation, and fever (80). Furthermore, FMT effectively reshapes the patient’s gut microbiota, and the sustained effects of these microbial changes have been observed in subsequent clinical follow-ups (81, 82).

Given the critical role of the gut microbiota in the human body, scientists are actively exploring optimal strategies for manipulating the gut microbiota to treat various diseases. The most common methods for such manipulation involve antibiotics or probiotic therapy. While antibiotics are life-saving, they can have adverse effects by eradicating beneficial microbiota and promoting antibiotic resistance in harmful bacteria (83). Scientific literature on the *in vivo* effects of probiotics suggests that these microorganisms can accumulate genetic mutations, transforming into ineffective or even harmful strains. However, the evolutionary capacity of probiotic microbiota can also serve as a novel therapeutic strategy against various diseases (84). In comparison to treatments involving prebiotics and probiotics, FMT directly introduces fecal bacteria into the colon to restore the microbiota.

#### 3.2.1 Preparation and application of FMT

Fecal Microbiota Transplantation (FMT) stands out as a highly efficient therapeutic intervention for microbiota modulation within a confined clinical setting. The procedure entails the transfer of gut microbiota from a meticulously screened healthy donor to a recipient, employing various delivery methods such as oral capsules, enema, or infusion via a nasoenteric tube. The primary objectives of FMT encompass the establishment of colonization resistance, the production of beneficial metabolites, and the restoration of interactions with the mucosal immune system (85). Throughout the FMT process, its impact extends beyond the mere transfer of microbiota, encompassing the restoration of the mucosal immune system, the presence of potentially virulent fungi in the microbial solution, transgenic metabolic byproducts, and notably, the pivotal role played by short-chain fatty acids (86). The earliest recorded use of fecal suspensions dates back to the 4th century when traditional Chinese medicine practitioner Ge Hong employed a concoction known as “yellow soup” to treat food poisoning and severe diarrhea (87).

In the European Consensus Conference, a collaborative effort involving 28 experts from 10 countries produced practical guidelines on indications for FMT, donor selection, fecal suspension preparation, clinical management, and the essential requirements for implementing FMT centers (88). Emphasizing the evaluation of FMT, particular attention is directed towards the selection of healthy donors. Prospective donors undergo comprehensive medical interviews aimed at excluding potential risk factors, ensuring the absence of genetic disorders, autoimmune diseases, infectious diseases, diabetes, and gastrointestinal issues. Eligible donors must not have utilized hormones, antibiotics, or proton pump inhibitors within the preceding 3 months. Additionally, they should not have received vaccinations or other investigational drugs in the last 6 months. Furthermore, prospective donors should not manifest symptoms of anxiety or depression (89). Regarding laboratory preparation methods, two alternative procedures are available: fresh preparation (90) and frozen preparation (91). The first method requires processing donor feces within 6 hours after defecation to preserve anaerobic bacteria integrity. Approximately 30 to 50 grams of feces are diluted with saline solution (NaCl 0.9%). The frozen method involves freezing the fecal suspension with glycerol at -80 degrees Celsius. Glycerol is essential for maintaining bacterial vitality during freezing. On the day of infusion, the frozen fecal suspension should thaw at 37°C and be diluted with 0.9% sodium chloride to the desired volume. Frozen feces are crucial for establishing a fecal bank and providing feces on demand. After preparation, FMT administration can be carried out through various routes, broadly categorized into upper digestive tract methods (nasogastric tube, nasoduodenal tube, nasojejunal tube, and capsules) and lower digestive tract methods (enema, colonoscopy) (90). Detailed analysis indicates that combining FMT with a diverse microbiota from a healthy donor promptly elevates diversity to normal levels, enhances microbial networks, and enriches the core microbial community (92).

Contaminated microorganisms in donor feces may lead to severe adverse events. Therefore, the preparation of fecal microbiota transplantation (FMT) material must exclude ineligible human, animal, or biological samples. Ongoing screening is required for donor feces, even from known donors, to eliminate infectious pathogens (93). For enhanced traceability, fecal samples from donors should be stored at deep low temperatures for at least two years (94). Despite the low occurrence and mild nature of short-term adverse events associated with FMT (95), long-term safety assessments are essential. Additionally, governmental agencies must prioritize the establishment of appropriate and effective regulations for FMT to ensure the safety of patients and donors, promote related research, and prevent treatment misuse (96).

#### 3.2.2 Application of FMT in patients with T2DM

**Figure 3:**
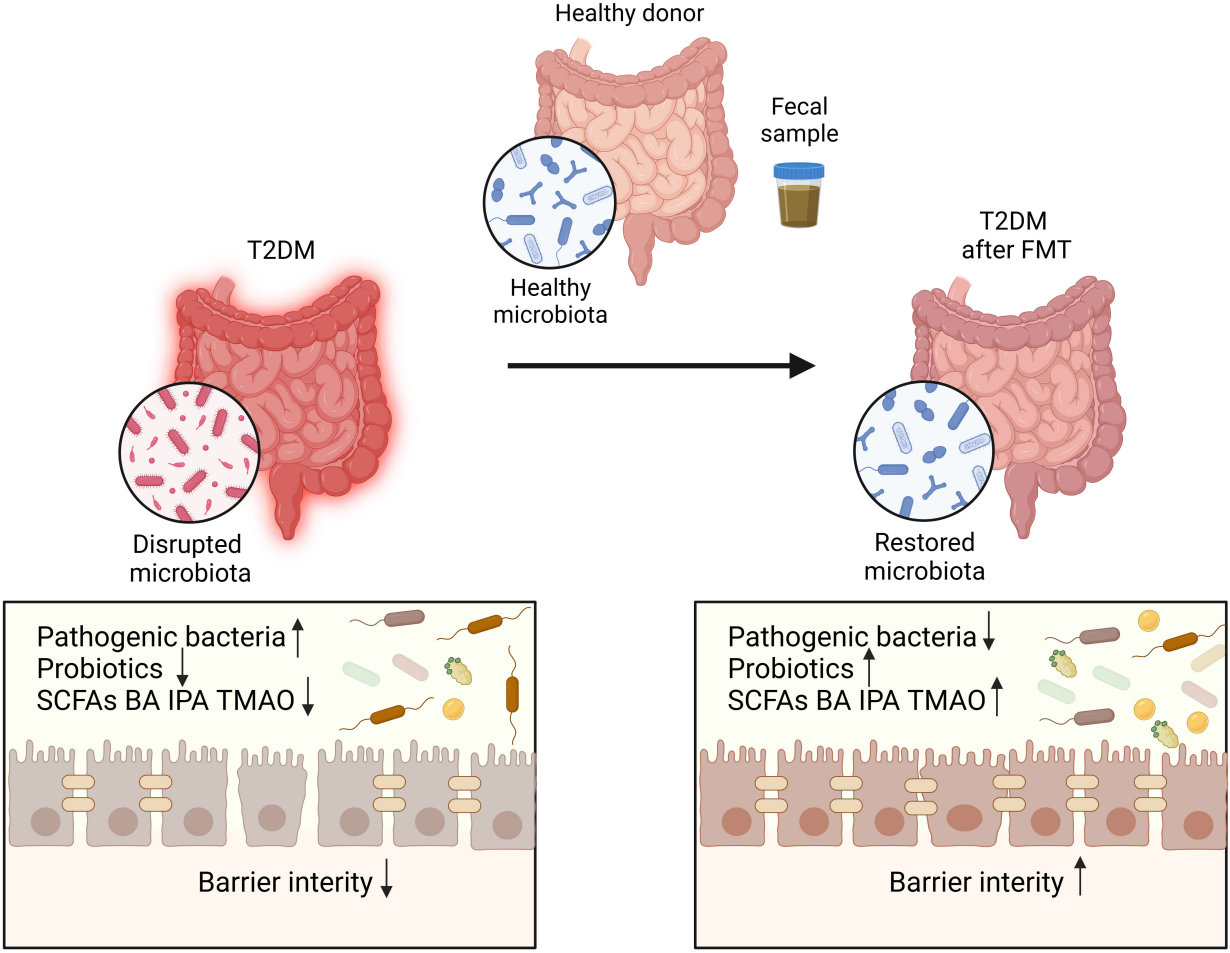
The disrupted microbiota in T2DM patients restored after FMT. Transplantation of healthy microbiota isolated from fecal sample of healthy donor can restore the disrupted microbiota of T2DM patients.

FMT has emerged as an effective strategy for treating T2DM (**Figure 3**, **Table 1**). Through the augmentation of short-chain fatty acid (SCFA) production, especially butyrate, Fecal Microbiota Transplantation (FMT) exhibits the capacity to mitigate intestinal permeability, thereby preserving the integrity of the epithelial barrier. Notably, the transplantation of F. prausnitzii has displayed favorable outcomes in transgenic models, underscoring its potential as a therapeutic approach for inflammation and diabetes through the restoration of the intestinal barrier’s structure and function (97). FMT elicits adaptive immune responses within the intestinal tract by activating the Toll-like receptor (TLR) pathway, leading to an accelerated synthesis of immunoglobulins that safeguard the intestinal mucosa (98). Furthermore, FMT demonstrates the ability to modulate the gut microbiota’s composition, influence the secretion of inflammatory cytokines, and regulate glucose and insulin sensitivity (99). Following FMT, there is an observed increase in the diversity of the gut microbiota, with research indicating its regulatory impact on the gut microbiota in type 2 diabetes mellitus (T2DM). This, in turn, results in enhanced glucose metabolism, improved insulin sensitivity, and a reduction in systemic inflammation.

**Table 1:** Routes and effects of FMT on different species with T2DM.

Application of FMT in T2DM			
Species	Route	Effects	Citation
Human	Oral FMT capsules in obese patients followed up to 26 weeks	Increased insulin sensitivity Increased gut microbial diversity Increased butyrate production	(81)
	500ml of faecal filtrate injected through gastroduodenal tube over 30 minutes, followed up to 6 weeks	Increased insulin sensitivity Increased butyrate-producing gut microbiota	(103)
	Single infusion of healthy lean donor faeces	Increased insulin sensitivity	(104)
	Microbiota prepared based on an automated purification system delivered endoscopically to the terminal ileum 2 times within every 3 months	Improved blood glucose control Reduced depressive symptoms and neuralgia	(105)
	Oral administration of autologous FMT capsules after dieting and weight loss, followed up to 6 months	Reduced weight regain Improved blood glucose control Altered gut microbiome composition and metabolic memory retention	(106)
	FMT combined with lifestyle intervention followed up to 24 weeks	Increased butyric acid-producing gut microbiota Decreased total lipoprotein cholesterol and LDL cholesFMT and lifestyle interventions up to 24 weeks of foterol Decreased liver stiffness	(108)
	Oral frozen FMT capsules followed up to 1 week	Improved HbA1c Altered gut microbiome composition	(109)
	Oral butyrate supplementation for 4 weeks after 500ml faecal filtrate injection through duodenal tube	Affects regulation of dopamine and serotonin transporters in the brain	(110)
Animal	Application of faecal solution from a healthy donor to a preclinical mouse model of diabetic kidney disease (DKD)	Prevention of weight gain	(10)
	Intragastric instillation of 0.3ml faecal suspension in T2D mice for 8 weeks	Stabilises and lowers blood glucose and improves glucose tolerance Improves insulin resistance and repairs injured islets Inhibits chronic inflammation in pancreatic tissue Reduces pancreatic beta cell apoptosis	(112)
	Diabetic rats injected with 80 ml of faecal microbiota solution once daily for 16 days	Reduced renal tubular interstitial damage Inhibited cholesterol homeostasis dysregulation	(113)
	Applied healthy donor faecal filtrate gavage to DKD mice for 14 days and executed 1 week later	Reduced blood glucose Reduced pathological damage	(114)
	Gavage of healthy rat faecal supernatant for 14 days in polycystic ovary syndrome (PCOS) rats	Improve androgen levels Affects insulin function	(115)
	Gavage of 0.2mL of faecal bacterial solution from normal glucose-tolerant individuals per db/db mouse	Altered the composition of the gut microbiome Reduced plasma glycolipid levels	(116)

In the context of gene expression in host bacteria, bacteriophages, as key regulators, exert a significant influence, even determining the survival of these bacteria. Prokaryotic viruses, particularly bacteriophages, represent the largest source of genomic material in feces, emphasizing their crucial role (100). Noteworthy observations in cases of recurrent Clostridioides difficile infection suggest that FMT, albeit in a limited number of patients, effectively alleviates diarrhea, pointing to the potential involvement of bacteriophages in preserving host health by regulating the composition and phenotype of the gut microbiota (101). In the study of metabolic syndrome, recipients of healthy donor allogeneic FMT showed a more similar fecal bacteriophage composition to donors compared to non-responders (102). Therefore, the potential of bacteriophage transplantation in relevant indications should not be overlooked.

A. Vrieze et al. (103) conducted a randomized controlled trial to assess the effects of fecal microbiota transplantation (FMT) on male patients with type 2 diabetes mellitus (T2DM). The study revealed that recipients of lean donor feces, facilitated through duodenal infusion (allogeneic transplantation), demonstrated improved insulin sensitivity and increased microbial diversity after six weeks compared to patients undergoing autologous transplantation (self-feces). The most notable changes in microbial composition included an increase in Roseburia intestinalis and Eubacterium hallii, both known producers of butyrate. In a study by Kootte et al. (104), enhanced insulin sensitivity was noted among individuals with obesity and metabolic syndrome who underwent Fecal Microbiota Transplantation (FMT) from lean donors. A study conducted by Allegretti et al. (81) delved into the impacts of FMT administered through oral capsules on obese patients. The findings revealed heightened insulin sensitivity, increased diversity in gut microbiota, and elevated butyrate production post-FMT. This investigative work lays the groundwork for future randomized controlled trials that explore the application of FMT or specific microbial combinations as potential interventions for obesity, metabolic syndrome, diabetes, and other endocrine disorders. In a case study, FMT (administered twice within three months) successfully alleviated blood glucose control, reduced depressive symptoms, and relieved neuropathic pain in a 46-year-old female diabetes patient (105). Additionally, no adverse reactions were observed. In a randomized controlled trial, it was observed that the application of autologous Fecal Microbiota Transplantation (FMT) following diet-induced weight loss preserved favorable metabolic attributes in the study participants (106). Particularly noteworthy was the finding that FMT sourced from obese donors resulted in swift weight gain, highlighting a potential correlation between gut microbiota, obesity, and insulin resistance (107). In fact, repetitive FMT in obese patients with T2DM increased the level and duration of microbial engraftment. Combining lifestyle interventions with FMT induced more favorable changes in recipients’ microbiota, improving lipid profiles and liver stiffness (108). Although another study did not find this impact on sugar metabolism with donor FMT, slight improvements in HbA1c post-FMT were noted. Regional differences among study populations and variations in FMT administration routes should be considered. Additionally, differences in transplanted microbial species, donor and recipient selection, and concurrent changes in microbiota and lifestyle are important factors to take into account (109). Furthermore, gut microbiota may produce metabolites that alter human behavior directly through the blood-brain barrier or indirectly by regulating gut-autonomic nerve activity, leading to changes in satiety and mood. Hence, Fecal Microbiota Transplantations (FMTs) may exert their influence on the gut-brain axis, modulating metabolites linked to emotions and satiety, pivotal factors in insulin resistance development. The metabolic profile of FMT donors might similarly impact the gut-brain axis in human recipients, potentially steering gut microbiota to regulate dopamine and serotonin transporters to the brain (110). Furthermore, allogeneic FMT derived from donors with metabolic syndrome feces led to reduced insulin sensitivity in metabolic syndrome recipients compared to those utilizing donors post-Roux-en-Y gastric bypass surgery (111). It is essential to highlight that these studies did not monitor blood glucose levels during the intervention, and not all participants responded to FMT (104).

In an animal experiment, Wang et al. (112) demonstrated that the reconstruction of the microbiota in type 2 diabetes (T2D) mice through FMT could reverse insulin resistance. In a groundbreaking study, Hu et al. (113) initially indicated that FMT alleviated tubulointerstitial damage in diabetic rats by mediating disruptions in cholesterol balance. Recently, researchers discovered in a preclinical mouse model of diabetic kidney disease (DKD), mirroring changes in human DKD, that FMT prevented weight gain, reduced albuminuria, mitigated local intestinal inflammation, and improved insulin resistance, providing new evidence for the role of FMT in diabetic patients (10). Moreover, Shang et al. (114) recently reported that Fecal Microbiota Transplantation (FMT), in comparison to empagliflozin treatment, exhibited a more pronounced reduction in blood glucose levels and ameliorated pathological damage in diabetic kidney disease (DKD) mice by modulating the gut microbiota. Additionally, FMT and Lactobacillus transplantation demonstrated improvements in testosterone levels and influenced insulin function in a rat model of polycystic ovary syndrome (115). Furthermore, FMT from a healthy donor significantly enhanced the composition of the gut microbiota in db/db mice, leading to reduced plasma glucose and lipid levels, notably with an elevation in the levels of the mucin-degrading bacteriophage A. muciniphila (116). Transfer of fecal samples from type 2 diabetes patients undergoing metformin treatment into germ-free mice resulted in enhanced glucose tolerance in the recipient mice (117). Similarly, investigations indicated that transferring fecal samples from type 2 diabetes patients treated with DPP-4 inhibitors into germ-free mice improved glucose tolerance, particularly in response to impairments induced by high-density lipoprotein cholesterol (118).

## 4 Discussion

Fecal Microbiota Transplantation (FMT) alone proves insufficient in effectively controlling blood glucose levels. Therefore, combining the modulation of gut microbiota with other established treatments for Diabetes Mellitus (DM), including lifestyle adjustments, medications such as metformin, sodium-glucose co-transporter-2 inhibitors (SGLT2i), GLP-1 receptor agonists, and lipid-lowering drugs like statins, yields improved metabolic parameters, ultimately alleviating the damage brought about by this complex pathophysiology (10).

When contemplating the transfer of microbiota from a donor to a recipient, it is crucial to account for potential variations in microbiota composition arising from disparate lifestyles between the donors and recipients. Therefore, if the recipient’s lifestyle fails to transition toward that of the donor post-FMT, the influence on microbiota composition is likely to attenuate over time (119). Various intervention studies convincingly indicate that diet influences microbiota composition, with changes occurring rapidly within days or weeks (120, 121). These changes can be robust, as demonstrated by studies collecting and performing autologous FMT during weight loss, maintaining the benefits of weight loss and blood sugar control even when specific microbiota characteristics and dietary habits are no longer adhered to (106). Conversely, individual microbiota composition can influence the response to dietary changes (122). These collective observations imply that dietary factors may independently influence the response to FMT by shaping the microbiota composition. Consequently, the standardization of dietary conditions during interventions has the potential to bolster the statistical robustness of FMT, eliminating a notable source of microbiota variability. Furthermore, it is noteworthy that exercise and physical fitness, although exerting a comparatively modest impact compared to diet, also appear to influence microbiota composition (123).

In summary, the introduction of healthy FMT has the potential to modify the gut microbiota, playing a protective role in the progression of T2DM. Nevertheless, a comprehensive understanding of the long-term outcomes of FMT in this context requires further elucidation (124). The imperative moving forward involves the conduct of additional high-quality prospective studies to furnish comprehensive and enduring safety and efficacy data, crucial for informing the clinical application of FMT in the management of T2DM.

Despite its limitations, FMT remains a crucial tool for investigating the causal relationships between the microbiota and a range of chronic diseases (125). To improve the effects of these studies, efforts should be directed towards further standardizing FMT procedures, including dosage response, administration modes, pre-processing, and whether to use fresh, frozen, or alternative pre-processing materials. Furthermore, there is a necessity for improved endpoint definition and more rigorous power calculations to enhance the precision of treatment effect estimation. To achieve consensus in this field, we propose the periodic convening of focused meetings, building upon recently initiated efforts (88).

## 5 Conclusions

The gut microbiota plays a pivotal role in both the initiation and progression of diabetes, influencing the response of intestinal and extraintestinal tissues to anti-diabetic interventions. This review aims to provide a comprehensive overview of current research on the involvement and mechanisms of the gut microbiota in anti-diabetic therapy, with a specific focus on ameliorating inflammation, regulating glucose homeostasis, and mitigating insulin resistance through the administration of anti-diabetic medications. Despite the intricacies of molecular pathways remaining somewhat elusive, advancements in sequencing technology and bioinformatics have facilitated a profound understanding of the intricate composition and abundance of metabolites originating from gut bacteria within biological samples. Current literature suggests the significant role of various compounds such as lipopolysaccharides (LPS), short-chain fatty acids (SCFAs), bile acids, and indoles in orchestrating the delicate balance of host metabolism. However, the specific bacterial strains responsible for inducing changes in these metabolites and the feasibility of culturing these strains *in vitro* remain uncertain and warrant further exploration. Additionally, the intricate mechanisms through which altered metabolites exert their regulatory effects on host metabolism need to be elucidated. It is imperative to acknowledge that a substantial portion of insights gleaned thus far emanates from animal experiments. Therefore, a critical challenge is discerning which observed phenotypes in animal models are pertinent to humans and can be faithfully replicated in human subjects. Consequently, an intensified focus on conducting more clinical trials is paramount to elucidate the potential therapeutic efficacy of gut bacteria and their derived metabolites in the context of diabetes.
