# Corrigendum: The role of fecal microbiota transplantation in type 2 diabetes mellitus treatment

**DOI:** 10.3389/fendo.2025.1555601

**Published:** 2025-01-27

**Authors:** Huimei Wang, Shuo Li, Luping Zhang, Nan Zhang

**Affiliations:** Department of Gastroenterology, The First Hospital of Jilin University, Changchun, China

**Keywords:** type 2 diabetes mellitus, fecal microbiota transplantation, gut microbiota, dysbiosis, metabolites

In the published article, there was an error in the order of the figures as published. The content of the figures is correct, but the sequence was not in order: Figure 3 should have appeared as **Figure 1**, Figure 1 should have appeared as **Figure 2**, and Figure 2 should have appeared as **Figure 3**. The corrected figures and their captions appear below.

**Figure 1:**
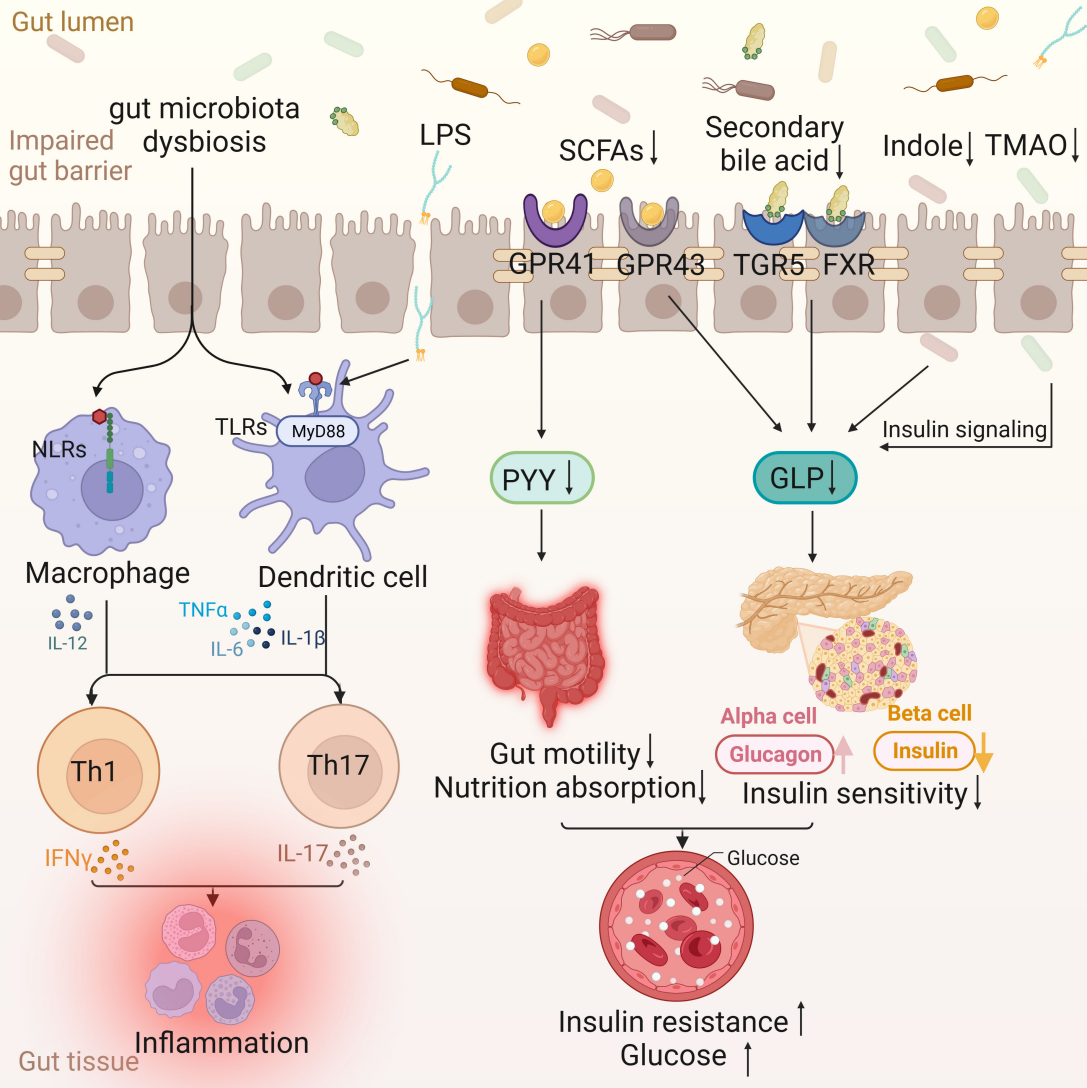
Intestinal changes in T2DM. The disordered gut microflora enters the gut tissue through the impaired gut barrier, activating the nucleotide oligomerization domain (NOD)-like receptors (NLRs) signaling pathway of macrophages and the MyD88-dependent TLRs signaling pathway of dendritic cells, and jointly activating Th 1 and Th 17, leading to the occurrence of inflammation. Lipopolysaccharides (LPS) is also involved in inflammation through Toll-like receptors (TLRs). The decrease of Short-chain fatty acids (SCFAs) leads to the decrease of peptide YY (PYY) through G protein-coupled receptor (GPCR) 41, which in turn leads to the decrease of gut motility and nutrient absorption function. At the same time, the decrease of SCFAs, secondary bile acids, indole and Trimethylamine N-Oxide (TMAO) can lead to the decrease of glucagon-like peptide (GLP) and insulin sensitivity through different ways, and then lead to insulin resistance and blood sugar increase.

**Figure 2:**
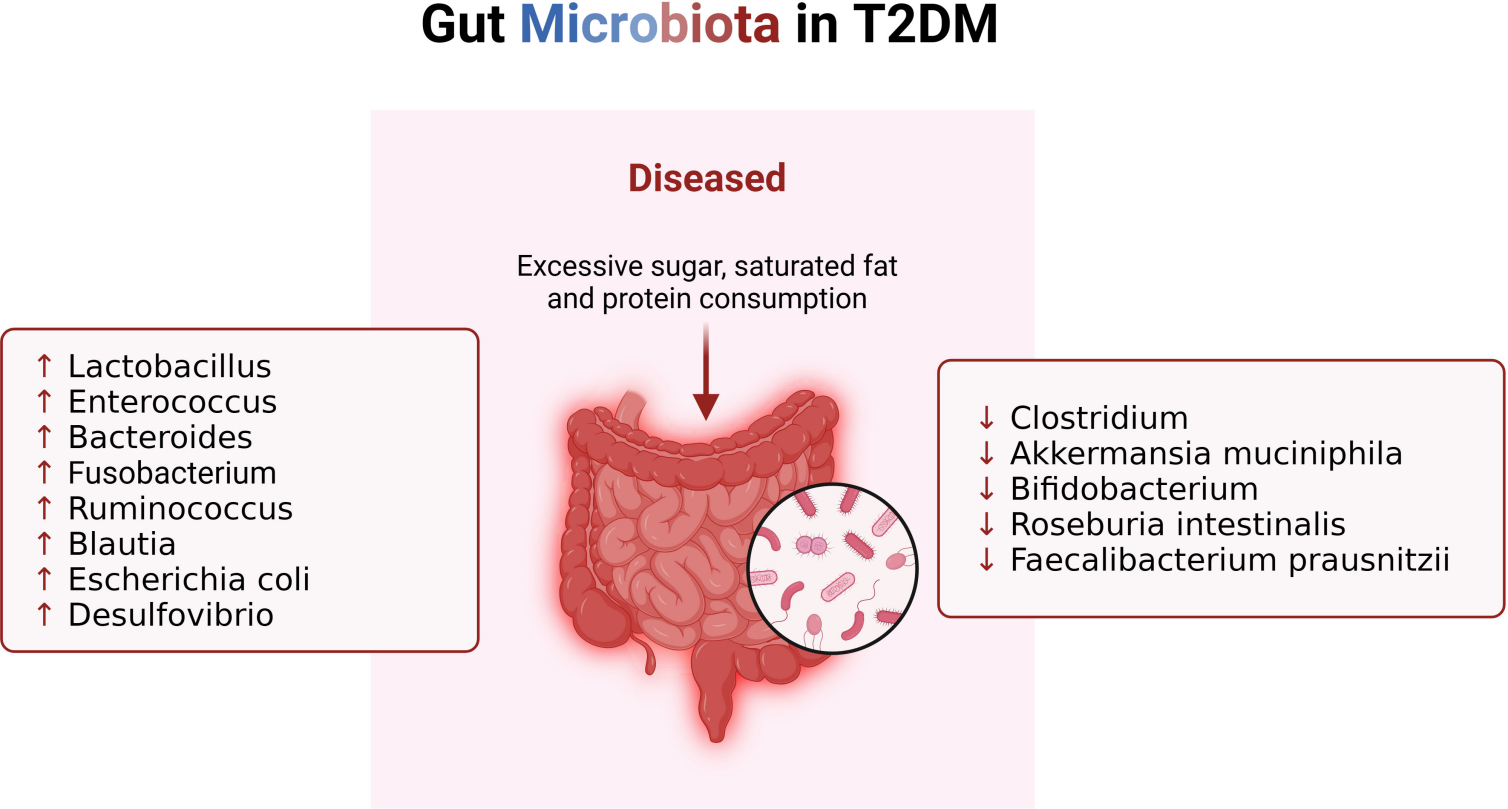
Changes of gut microbiota in patients with T2DM. When T2DM patients consume excessive sugar, saturated fat and protein, their gut microbiota changes, resulting in imbalance of immune system.

**Figure 3:**
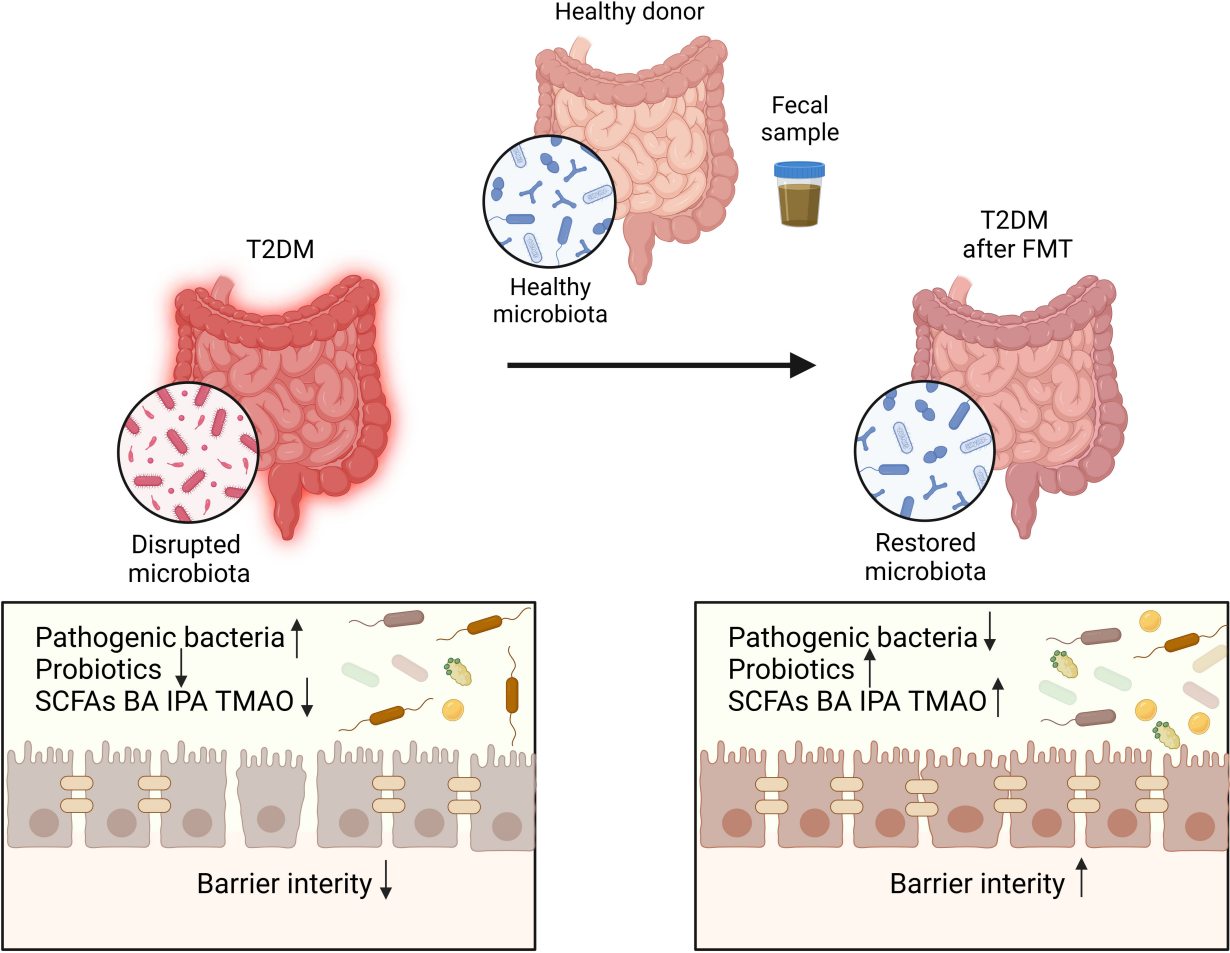
The disrupted microbiota in T2DM patients restored after FMT. Transplantation of healthy microbiota isolated from fecal sample of healthy donor can restore the disrupted microbiota of T2DM patients.

The authors apologize for this error and state that this does not change the scientific conclusions of the article in any way. The original article has been updated.

